# Piezo1 Channel Activators Yoda1 and Yoda2 in the Context of Red Blood Cells

**DOI:** 10.3390/biom15081110

**Published:** 2025-08-01

**Authors:** Min Qiao, Reetta Penttinen, Ariel Coli, Nicoletta Murciano, Felix M. Maurer, Christian Wagner, Maria Giustina Rotordam, Lars Kaestner

**Affiliations:** 1 Theoretical Medicine and Biosciences, Medical Faculty, Saarland University, 66421 Homburg, Germany; min.qiao@uni-saarland.de (M.Q.); reetta.penttinen@nanion.de (R.P.); 2 Dynamics of Fluids, Experimental Physics, Faculty of Natural Science and Technology, Saarland University, 66123 Saarbrücken, Germany; ariel.coli@uni-saarland.de (A.C.); felixmilan.maurer@uni-saarland.de (F.M.M.); christian.wagner@uni-saarland.de (C.W.); 3 Nanion Technologies, 80339 Munich, Germany; nicoletta.murciano@nanion.de (N.M.); giustina.rotordam@nanion.de (M.G.R.); 4 Physics and Materials Science Research Unit, University of Luxembourg, 1511 Luxembourg, Luxembourg

**Keywords:** erythrocytes, Piezo1, Yoda1, Yoda2, mechanosensitive ion channel, automated patch-clamp, intracellular calcium, live cell imaging, membrane potential, MBE-method

## Abstract

Piezo1 is a mechanosensitive non-selective cation channel. Genetic alterations of the channel result in a hematologic phenotype named Hereditary Xerocytosis. With Yoda1 and, more recently, Yoda2, compounds to increase the activity of Piezo1 have become available. However, their concrete effect depends on the nano environment of the channel and hence on the cell type. Here we compare the potency of Yoda1 and Yoda2 in red blood cells (RBCs). We investigate the effect of the compounds on direct channel activity using automated patch clamp, as well as the secondary effects of channel activation on signalling molecules and cellular response. In terms of signalling, we investigate the temporal response of the second messenger Ca^2+^, and in terms of cellular response, the activity of the Gárdos channel. The opening of the Gárdos channel leads to a hyperpolarisation of the RBCs, which is measured by the Macey–Bennekou–Egée (MBE) method. Although the interpretation of the data is not straightforward, we discuss the results in a physiological context and provide recommendations for the use of Yoda1 and Yoda2 to investigate RBCs.

## 1. Introduction

Piezo1 is a large, mechanically activated ion channel that plays a key role in cellular mechanotransduction and, in terms of cellular response, in converting mechanical stimuli into electrochemical signals across a wide range of cell types [1,2,3,4,5]. It acts as a non-selective cation channel permeable to Ca^2+^; mechanical forces trigger its opening, leading to Ca^2+^ influx and the activation of downstream signalling pathways [6]. In human red blood cells (RBCs), Piezo1 is endogenously abundant and plays a crucial role in cell volume regulation by activating the Ca^2+^-sensitive Gárdos channel and potentially other effectors [7,8,9].

Beyond its physiological functions, Piezo1 dysfunction is associated with Hereditary Xerocytosis, a rare haemolytic but mostly compensated anaemia caused by gain-of-function mutations in Piezo1 and characterised by dehydrated RBCs [10,11,12,13,14].

Although Piezo1 is directly gated by mechanical forces, its activation is strongly influenced by the physical and chemical properties of the surrounding membrane. Even in the absence of cytoskeletal constraints, membrane features such as curvature, lipid composition, and cholesterol content can modulate channel sensitivity by altering local tension and bilayer stress [15,16,17,18,19,20]. These properties help explain the diversity in Piezo1 activity across different cell types and environments.

Small molecules such as Yoda1 and its analogue Yoda2 have emerged as important pharmacological tools for modulating Piezo1 activity. Rather than bypassing mechanical activation, these compounds stabilise the channel in open or pre-open conformations, thereby lowering the threshold for mechanical activation [21,22]. Their effects are context-dependent and shaped by factors such as membrane architecture, curvature, and lipid environment [17,20,23].

In this study, we investigate the pharmacological activation of Piezo1 in human RBCs using Yoda1 and Yoda2. By integrating high-throughput electrophysiology, calcium measurements, and assays of Gárdos channel activity, we compare their potency and characterise downstream responses in this unique native cell type. These findings offer new insight into the context-dependent pharmacology of Piezo1 activators and their applicability in RBCs.

## 2. Materials and Methods

### 2.1. Blood Collection and Preparation

All measurements were performed on freshly drawn venous blood obtained from healthy donors as approved by the ethics vote of the “Ärztekammer des Saarlandes” (reference number 51/18). For Ca^2+^ and membrane potential measurements, RBCs were washed three times in Tyrode (see Section 2.3 for solution composition) or 0.9% NaCl solution, respectively. RBCs were centrifuged at 3880 RCF (for membrane potential measurements) or 500 RCF (for Ca^2+^ measurements) for 5 minutes. The plasma supernatant was removed, and the RBC pellet was diluted with the respective solution. This washing step was repeated twice with gentle mixing of the pellet, and a subsequent centrifugation was performed between each wash. For the patch clamp measurements, whole blood was diluted in the external recording solution (see Section 2.2 for solution composition) without any additional processing. Blood samples for all experiments were used within 3 hours of withdrawal.

### 2.2. Automated Patch Clamp Recordings

Whole-cell patch clamp recordings were performed using the SyncroPatch 384 (Nanion Technologies, Munich, Germany) as previously described [24]. Experiments were conducted at room temperature using NPC-384 single-hole microchips with resistances ranging from 6 to 10 MΩ. Recording solutions contained (in mM) 110 KF, 10 KCl, 10 NaCl, 10 EGTA, and 10 HEPES, pH 7.2 (KOH) (internal); and 140 NaCl, 4 KCl, 2 CaCl_2_, 1 MgCl_2_, 5 D-glucose monohydrate, and 10 HEPES, pH 7.4 (NaOH) (external). Data acquisition was conducted using PatchControl384 v3.2.2 (Nanion Technologies, Munich, Germany), sampled at 10 kHz.

Piezo1-mediated currents were elicited using a voltage ramp protocol ranging from −100 mV to +80 mV over 450 ms, applied every 10 s, with a holding potential set at −30 mV. After establishing a stable baseline current in the external solution, cells were exposed to increasing concentrations of either Yoda1 or Yoda2 (Tocris Bioscience, Bristol, UK) in a cumulative manner: 41 nM, 144 nM, 442 nM, 1.33 μM, 4 μM, and 8 μM. To block channel activity, 30 μM GdCl_3_ (Sigma-Aldrich, Merck KGaA, Taufkirchen, Germany, a non-selective ion channel inhibitor, was subsequently applied.

RBCs exhibiting a seal resistance greater than 0.5 GΩ were selected for analysis. RBCs were classified as responders if the mean current amplitude in the presence of the compound exceeded the mean current at baseline conditions by more than three times the standard deviation of the baseline (3σ). For EC_50_ determination, current amplitudes at each concentration were normalised to the baseline current, and dose–response curves were fitted using the Hill equation.

### 2.3. Calcium Imaging and Flow Cytometry

Experiments for the intracellular Ca^2+^ detection of RBCs were performed in Tyrode solution containing (in mM) 130 NaCl, 5 KCl, 5 glucose, 10 HEPES, 1 MgCl_2_, and 1.5 CaCl_2_. The pH was adjusted to 7.40 with NaOH. All salts were of analytical grade. The cells were loaded with Fluo-4 AM (Molecular Probes, Eugene, OR, USA) at a concentration of 5 μM for 1 hour at 37 °C. Then, the cells were washed three times with Tyrode solution.

Flow cytometry measurements were performed on LSRFortessa (Becton Dickinson, Franklin Lakes, NJ, USA). Washed RBCs from 50 μL of blood were loaded with Fluo-4 as previously described [25] and washed again. For flow cytometry analysis, 10 μL of cell suspension was added to 700 μL of designed buffer and transferred to 5 mL round-bottom polystyrene test tubes (Corning Inc., Corning, NY, USA). For each sample, data recording started 1 minute after the cells were in the designed buffer for 10 minutes. The results were assessed using FlowJo (Becton Dickinson, Franklin Lakes, NJ, USA).

For calcium imaging, RBCs from 10 μL of blood were washed three times in Tyrode solution. Then, cells were loaded with Fluo-4 as described above and washed again. For calcium images, 50 μL of cell suspension was added to 450 μL of Tyrode solution and transferred to µ-Dishes (ibidi, Munich, Germany). Ca^2+^ images were acquired by a Leica STELLARIS 5 microscope (Leica Microsystems, Mannheim, Germany) with an HC PL APO CS2 63×/1.40 oil objective (Leica Microsystems, Mannheim Germany). Time-lapse acquisition was performed with a 488 nm laser recording a z-stack every 30 s, starting 90 s after the compounds were applied to the cells.

### 2.4. Membrane Potential Measurements

The membrane potential of RBCs was measured using a method originally described by Macey et al. [26], subsequently refined by Poul Bennekou, e.g., [27], and later optimised by the group of Stéphane Egée, e.g., [28]. As such, the method is referred to as the MBE method after the three scientists who most contributed to it [29]. For each experiment, a 2 mL microcentrifuge tube was prepared with 1 mL of Ringer solution containing (in mM) 153 NaCl, 2 KCl, and 4 CaCl_2_. All reagents were of analytical grade and dissolved in Milli-Q water. Protonophore carbonyl cyanide m-chlorophenyl hydrazone (CCCP) was added at a final concentration of 26 µM to increase the hydrogen permeability of membranes. The tube was placed in a 36 °C water bath, and a magnetic stir bar was added to ensure continuous mixing. A calibrated pH electrode (InLab Expert Pro pH electrode, Mettler-Toledo GmbH, Greifensee, Switzerland) was immersed in the solution to monitor pH changes in real time, with data acquisition and recording performed via software on a connected computer. One minute after the start of pH recording, 150 µL of packed RBCs was added to the tube. At 1.5 minutes following RBC addition, the test compound (either Yoda1 or Yoda2) was introduced at the desired concentration. The system was monitored for an additional 5 minutes to observe the effects of the compound on the RBCs. Finally, 80 µL of Triton X-100 (6% Triton X-100 in 3M NaCl solution) was added to lyse the RBCs in order to establish a reference condition corresponding to a membrane potential of 0 mV.

The membrane potential (V_m_) was calculated from the recorded pH data using the following equation:

V_m_ [mV] = −61.5 × (pH_val_ − pH_lys_),

where pH_val_ is the pH value measured at a specific time point, and pH_lys_ is the final pH value obtained after cell lysis.

### 2.5. Data Analysis and Statistics

DataControl 384 v3.2.1 (Nanion Technologies, Munich, Germany) was used for the analysis of automated patch clamp data. *n* represents the number of responding cells out of the total number of valid cells, and *N* indicates the number of independent NPC-384 chips. Microscopic images were processed by Fiji (ImageJ Release2.16.0). Membrane potential data were analysed using Excel (Office 365, Microsoft Corporation, Redmond, WA, USA) and MATLAB (R2023a, MathWorks, Natick, MA, USA). Statistical analysis was performed using Prism 10 (v10, GraphPad software, Boston, MA, USA). Throughout the paper, data are presented as mean values with error bars indicating the standard error of the mean (SEM). Origin (v2023b, OriginLab Corporation, Northampton, MA, USA) and Prism were used for the presentation of the data.

## 3. Results

### 3.1. Patch Clamp Recordings

High-throughput automated patch clamp recordings were conducted to compare the electrophysiological effects of the Piezo1 activators Yoda1 and Yoda2 in RBCs. Due to the heterogeneity of the RBCs, such as variability in cell age and ion channel expression, responses to different concentrations of Piezo1 agonists can vary among individual cells, complicating precise EC_50_ determination from single-dose exposures. To address this, a cumulative concentration approach was used, in which each cell was sequentially exposed to either increasing concentrations of the compound (Yoda1 or Yoda2) or the reference solution (external solution + 0.1% DMSO). This approach allowed the assessment of individual responsive cells across the entire concentration range, thereby increasing the number of data points per cell and improving the reliability of potency estimations.

**Figure 1:**
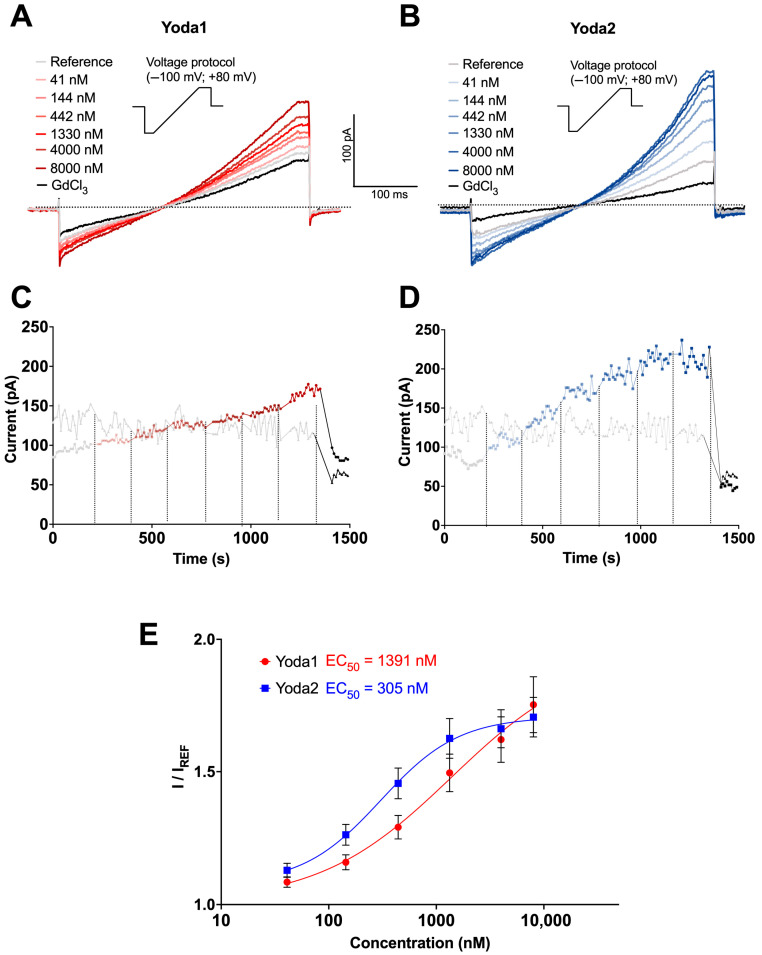
Comparative analysis of Piezo1 activators Yoda1 and Yoda2 using automated patch clamp. (**A**,**B**) Representative current ramp traces recorded from human RBCs. Voltage ramps from −100 mV to +80 mV (insets) were applied every 10 s. Cells were initially perfused with external solution (grey traces), followed by cumulative additions of Yoda1 (**A**; 41 nM, 144 nM, 442 nM, 1330 nM, 4000 nM, and 8000 nM; red traces) or Yoda2 (**B**; 41 nM, 144 nM, 442 nM, 1330 nM, 4000 nM, and 8000 nM; blue traces). Subsequently, 30 μM GdCl_3_ was applied to block Piezo1 channels (black traces). (**C**,**D**) Time-course plots of current amplitudes at +80 mV from representative cells exposed to cumulative applications of external solution (**C**,**D**, grey traces), increasing solutions of Yoda1 (**C**, red traces) or Yoda2 (**D**, blue traces), and GdCl_3_ (**C**,**D**, black traces). Dashed vertical lines indicate the time points of each solution addition. (**E**) Concentration–response curves for Yoda1 and Yoda2. Current amplitudes at +80 mV were normalised to the baseline current in the external solution (I/I_REF_). Data points represent mean ± SEM. Curves were fitted using the Hill equation to estimate the EC_50_s. These data were obtained from the subset of RBCs classified as responders, defined as RBCs in which the current amplitude in the presence of the compound at any concentration exceeded the baseline current by more than three times the standard deviation (3σ) of the baseline. The EC_50_ values were determined to be 1391 nM for Yoda1 (*n* = 35/172; *N* = 3) and 305 nM for Yoda2 (*n* = 35/171; *N* = 3), indicating a higher potency and efficiency of Yoda2 in activating the Piezo1 channel. Here, *n* represents the number of responding cells out of the total number of cells that met the preset quality control conditions, and *N* indicates the number of independent NPC-384 well plates.

**Figure A1:**
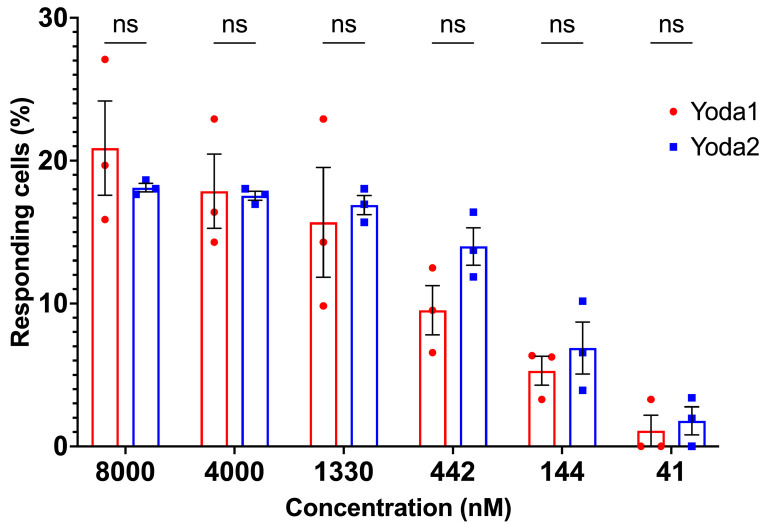
The responding cells at +80 mV of Piezo1 agonists Yoda1 and Yoda2 recorded using automated patch clamp. Yoda1: 8000 nM: *n* = 35/172; 4000 nM: *n* = 30/172; 1330 nM: *n* = 26/172; 442 nM: *n* = 16/172; 144 nM: *n* = 9/172; 41 nM: *n* = 2/172; *N* = 3; Yoda2: 8000 nM: *n* = 31/171; 4000 nM: *n* = 30/171; 1330 nM: *n* = 30/171; 442 nM: *n* = 24/171; 144 nM: *n* = 12/171; 41 nM: *n* = 3/171; *N* = 3. The bars are mean ± SEM, indicated in black; the circles are the data points from individual experiments. The fraction of cells responding to Yoda1 increased with concentration, mirroring the concentration–response relationship. In contrast, the fraction of cells responding to Yoda2 increased progressively with concentrations up to 1300 nM, after which current amplitudes plateaued or slightly decreased, hence no longer meeting the criteria for responding cells at the highest concentrations. This accounts for the variability between the total number of cells classified as responders across the full range of Yoda2 concentrations (*n* = 35) and the number of cells responding at the highest concentration (*n* = 31). *n* represents the number of cells at any tested concentration for a given experimental condition out of the total number of valid cells, and *N* indicates the number of independent experiments.

Measurements are presented in Figure 1 with analysis at membrane potentials of +80 mV. Under control conditions, where only the reference solution was applied, currents remained stable throughout the recording period (Figure 1C,D, grey traces), confirming that the mechanical stress generated by fluid exchange alone was insufficient to activate the channels. GdCl_3_ could block Piezo1 channel activity completely. In contrast, both Yoda1 (Figure 1A,C, red traces) and Yoda2 (Figure 1B,D, blue traces) elicited concentration-dependent currents, consistent with Piezo1 activation. Figure 1C,D show the currents at +80 mV. Moreover, Yoda1 induced a gradual increase in current amplitude across all tested concentrations (Figure 1A,C), while Yoda2 evoked a more step-like current increase, with the current plateauing at concentrations above 1 μM (Figure 1B,D). This plateau suggests that Yoda2 may reach channel saturation at lower concentrations than Yoda1. The analysis is based exclusively on responding cells. The statistics of the responding cells are presented in Appendix A (Figure A1).

Normalised concentration–response curves (Figure 1E) confirmed the higher potency of Yoda2, with an estimated EC_50_ of 305 nM compared to 1391 nM for Yoda1. At sub-micromolar concentrations, Yoda2 elicited a greater response than Yoda1 and plateaued at around 4 μM. However, at the highest concentration, this trend was reversed: Yoda1 did not plateau and exhibited a more variable response, likely due to its solubility issues. The fraction of responding cells followed a similar pattern, being higher, though not statistically significant, for Yoda2 at sub-micromolar concentrations and reversed at higher concentrations. The response to Yoda2 was overall more consistent than for Yoda1. In general, the fraction of responding cells closely mirrored the dose–response relationship observed for the two compounds (Figure A1).

These results demonstrate that both Yoda1 and Yoda2 activate Piezo1 channels in RBCs in a dose-dependent manner, with Yoda2 exhibiting higher potency and efficacy. These findings position Yoda2 as a valuable pharmacological tool for modulating Piezo1 activity in RBCs, particularly in patch clamp assays where its stronger activation profile enhances detection sensitivity and functional characterisation.

### 3.2. Calcium Measurements

**Figure 2:**
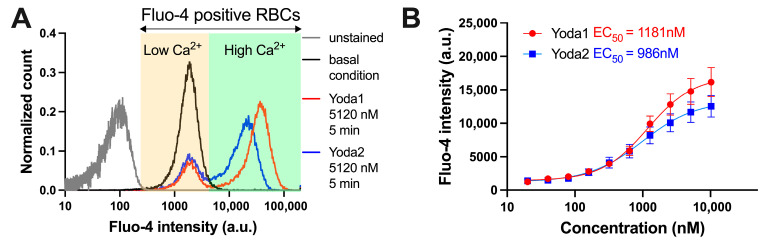

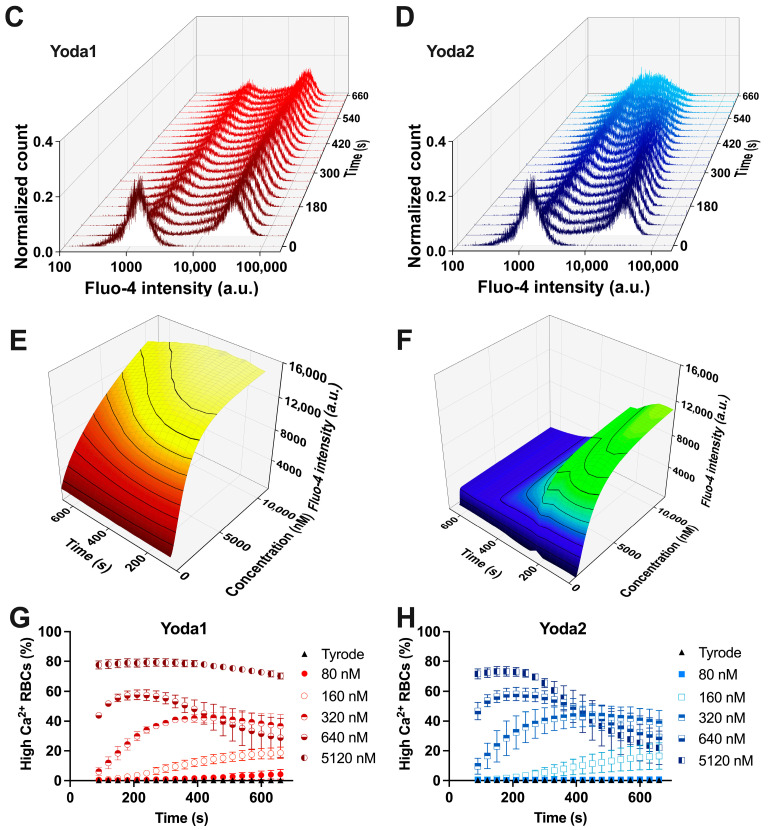
Comparison of action of Yoda1 and Yoda2 on the free Ca^2+^ in healthy red blood cells (RBCs). (**A**) Representative normalised histograms of Fluo-4 intensity of unstained RBCs (gray), Fluo-4-stained RBCs in Tyrode solution (black), Fluo-4-stained RBCs with Yoda1 stimulation (red), and Fluo-4-stained RBCs with Yoda2 stimulation (blue). (**B**) Maximum Fluo-4 intensity during recording for each compound was used to construct fitted curves from the Hill equation, and the respective EC_50_ values are indicated. (**C**,**D**) Representative normalised histograms of Fluo-4 intensity of RBCs after 5120 nM Yoda1 (**C**) and Yoda2 (**D**) stimulation. Histograms for all concentrations applied are provided in Appendix B. (**E**,**F**) Three-dimensional presentation of fitted curves from the Hill equation by using the average Fluo-4 intensity of RBCs after Yoda1 (**E**) and Yoda2 (**F**) stimulation over time. (**G**,**H**) Percentage of the high Ca^2+^ RBC population over time after Yoda1 (**G**) and Yoda2 (**H**) stimulation. Data are shown as means ± SEM in panels (**B**,**G**,**H**). (*N* = 3 independent experiments with at least 30,000 cells per time interval).

**Figure 3:**
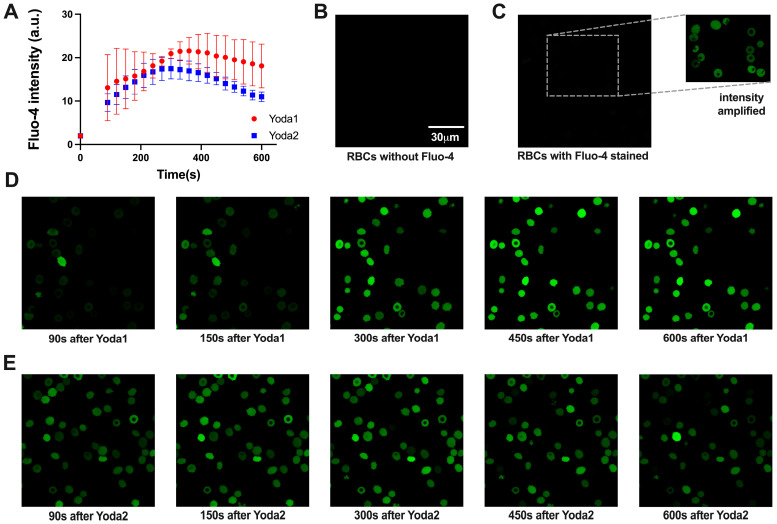
Exemplified microscopy analysis of Ca^2+^ signalling of healthy red blood cells (RBCs) with 1.28 μM Yoda1 or Yoda2 stimulation. (**A**) The cellular Ca^2+^ response was plotted as average Fluo-4 intensity over time for all RBCs *(N* = 2 independent experiments, each of *n* > 50 cells). (**B**–**E**) Confocal images of RBCs at different conditions: (**B**) unstained RBCs; (**C**) Fluo-4-stained RBCs in Tyrode solution; the inset is intensity amplified to visualise the cells; (**D**) Fluo-4-stained RBCs after Yoda1 stimulation with temporal information indicated below the images; (**E**) Fluo-4-stained RBCs after Yoda2 stimulation with temporal information indicated below the images. The scale bar given in (**B**) is valid for all images, and all images had the same acquisition settings and are displayed in the same contrast settings, except the insert in panel (**C**).

**Figure A2:**
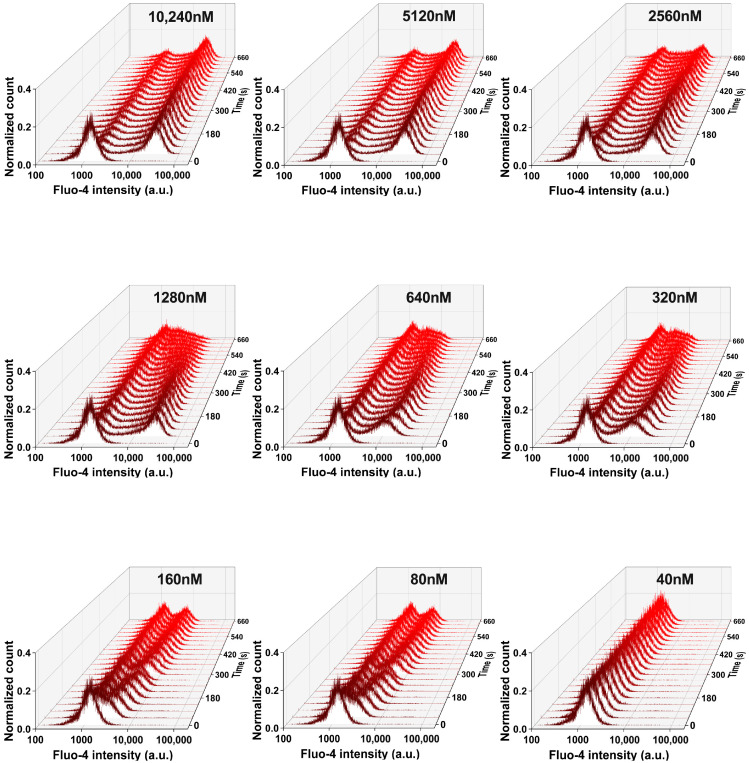
Representative normalised histograms of Fluo-4 intensity of RBCs after Yoda1 stimulation over time. The concentration of Yoda1 is indicated in each panel.

**Figure A3:**
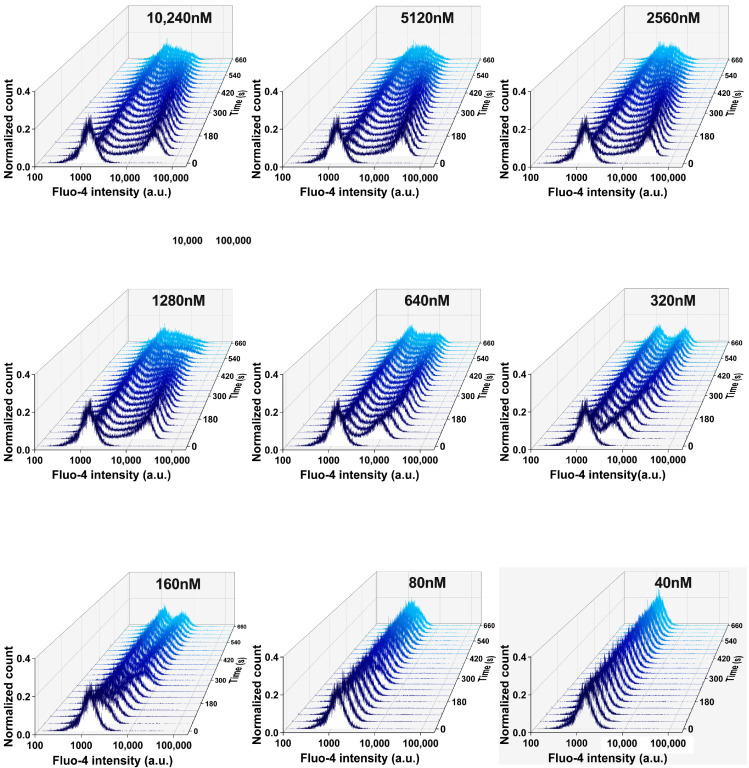
Representative normalised histograms of Fluo-4 intensity of RBCs after Yoda2 stimulation over time. The concentration of Yoda2 is indicated in each panel.

**Figure A4:**
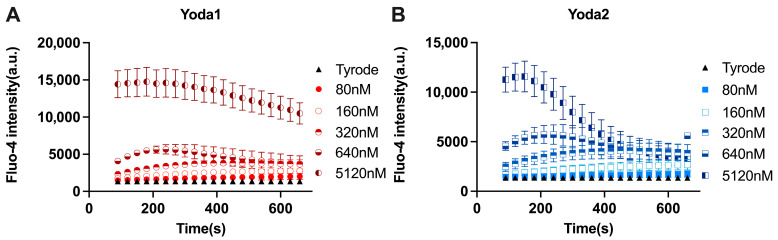
Average Fluo-4 intensity of all red blood cells (RBCs) over time after (**A**) Yoda1 and (**B**) Yoda2 stimulation. Data are shown as means ± SEM. (*N* = 3 independent experiments, *n* > 30,000 cells per time interval).

As a non-selective cation channel, the activation of Piezo1 leads to Ca^2+^ entry, increasing intracellular Ca^2+^. When Fluo-4-stained RBCs were challenged with Yoda1 or Yoda2, a group of RBCs showed increased Fluo-4 intensity, indicating an increased intracellular Ca^2+^ concentration (Figure 2). The increased Ca^2+^ induced by Yoda1 or mechanical stress can be significantly inhibited by GsMTx4, a Piezo1 channel inhibitor [30,31]. Yoda2-induced high Ca^2+^ RBCs had a lower average Fluo-4 intensity than those induced by Yoda1 stimulation (Figure 2). The full characterisation of the cells with all Yoda concentrations was performed in flow cytometry (Figure 2 and Appendix B, Figure A2, Figure A3 and Figure A4). In addition, some of the measurements were performed by microscopic Ca^2+^ imaging (Figure 3) in order to test whether the shear and putative mechanical stress that RBCs experience in the flow cytometer had an influence on the measurements. Flow cytometry and confocal imaging gave consistent results. The average Fluo-4 intensity of high Ca^2+^ RBCs decreased over time when the Yoda1 or Yoda2 concentration was above 1.28 μM. In addition, the high Ca^2+^ concentration was maintained for longer and decreased more slowly in Yoda1-induced high Ca^2+^ RBCs compared to Yoda2 (Figure A2 and Figure A3, respectively). When RBCs were challenged with 640 nM Yoda1 or Yoda2, the average Fluo-4 intensity of both samples initially increased and then decreased. On the other hand, when RBCs were challenged with 320 nM Yoda2, the Fluo-4 intensity of the high Ca^2+^ population increased over time, whereas the Yoda1-induced high Ca^2+^ population showed first an increased Fluo-4 intensity, followed by a decreased Fluo-4 intensity, after approximately 150 s. When RBCs were stimulated with a lower dose of Yoda1 or Yoda2, the Fluo-4 intensity of the high Ca^2+^ population increased over time, and the RBCs showed no obvious response to Yoda2 at 80 nM or Yoda1 at 40 nM (Figure A2 and Figure A3). Additionally, the percentage of the high Ca^2+^ population exhibited a similar trend to the average Fluo-4 intensity (Figure 2G,H).

Due to changes in the intracellular Ca^2+^ concentration of the high Ca^2+^ population, the average Fluo-4 intensity of all RBCs changes over time. The fitted Hill equation and the extracted EC_50_ are changing over time (Figure 2E,F). The maximum average Fluo-4 intensity of all RBCs in each experiment was extracted and used to fit the Hill equation. The S-curve of Yoda2 has a lower plateau than that of Yoda1. The EC_50_ of Yoda2 is 986 nM, which is smaller than that of Yoda1 (1181 nM) (Figure 2B).

### 3.3. Membrane Potential Measurements

**Figure 4:**
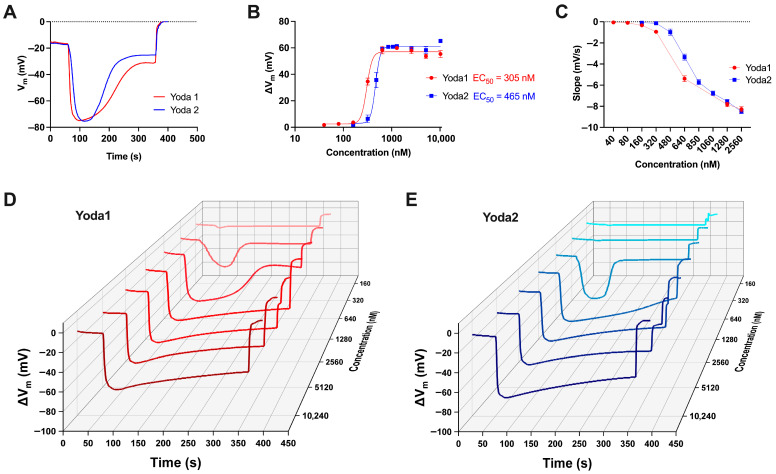
The comparison of Piezo1 activators Yoda1 and Yoda2 in a population of healthy red blood cells (RBCs) using the MBE method. (**A**) Representative recordings of membrane potential (V_m_) changes in RBCs upon exposure to 640 nM Yoda1 (red) and Yoda2 (blue). Recording starts at the resting membrane potential (V_rest_) of RBCs. The trace shows the addition of the reagent—Yoda1 or Yoda2—as well as the addition of Triton-X at the end so as to lyse the cells (reference point 0 mV). (**B**) Dose–response curves for Yoda1 and Yoda2, showing the effect of varying concentrations of the reagents. For each concentration, the magnitude of membrane hyperpolarisation ΔV_m_ is obtained from the average of two or more measurements from different donors. The Hill equation is used to fit curves to the data. The respective EC_50_ values for Yoda1 and Yoda2 are indicated on the plot. (**C**) Representation of the dose dependency of the slope in RBC samples after the addition of reagents. The slope is determined by identifying the membrane potential values at one-third and two-thirds of the range between the resting membrane potential and the peak membrane potential. Specifically, the slope is calculated as (V_b_ − V_a_)/(T_b_ − T_a_), where V_a_ and V_b_ are the membrane potentials at one-third and two-thirds of the range, respectively, and T_a_ and T_b_ are the corresponding time points. This reflects the rate of hyperpolarisation within the central portion of the response curve. (**D**,**E**) Three-dimensional waterfall plot displaying representative traces of membrane potential changes in RBCs following exposure to varying concentrations of Yoda1 and Yoda2. Data are expressed as mean ± SEM.

Membrane potential measurements revealed the effects of the Piezo1 activators Yoda1 and Yoda2 on RBCs. Each RBC sample was exposed to different concentrations of the activators, ranging from 10.24 μM to 160 nM, in order to assess the impact of the reagents and to determine key analytical parameters such as the EC_50_. Representative recordings that illustrate changes in membrane potential are shown in Figure 4A.

**Figure 5:**
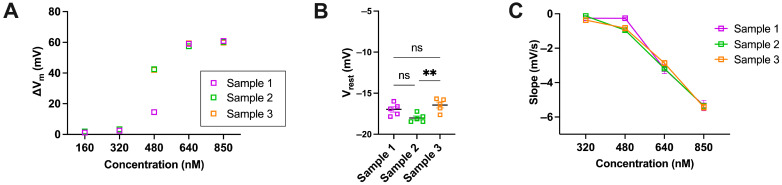
(**A**–**C**) Graphs representing the biological variability of samples as a means to determine that variation of results during experiments is sample-dependent and not mechanical error. Samples from three donors were measured under identical conditions to ensure consistency in the results. (**A**) Scatter plot showing ΔV_m_ of red blood cells (RBCs) exposed to five different concentrations of Yoda2. (**B**) Bar graph comparing the V_rest_ of three different donors. ** indicate a significance *p* < 0.01 and ns *p* > 0.05. (**C**) Representation of the dose dependency of the slope in RBC samples after addition of different concentrations of Yoda1 and Yoda2. Statistical significance in the graphs was determined using ordinary one-way ANOVA. Data are expressed as mean ± SEM. Significant differences between groups were considered at *p* < 0.05. All experiments were conducted under identical conditions and on the same day in order to ensure that observed variability arose solely from biological differences between samples rather than external or mechanical inconsistencies.

These traces indicated the timing of reagent addition, the magnitude of membrane hyperpolarisation (ΔV_m_, defined as the difference between peak and resting membrane potentials), and the final addition of Triton X-100, which lysed the RBCs and established a reference condition corresponding to a membrane potential of 0 mV. It is known that the hyperpolarisation is largely inhibited if RBCs are pretreated with the Piezo1-specific inhibitor GsMTx4 [32]. Figure 4B presents the dose–response curves for both Yoda1 and Yoda2, along with the calculated EC_50_ values, which were 305 nM and 465 nM, respectively. The data indicated that while both compounds activated Piezo1 channels in RBCs at varying concentrations, there were notable differences in the cellular responses elicited by each. To quantify the rate of membrane hyperpolarisation, the slope of the membrane potential change was extrapolated from the dataset using MatLab and subsequently plotted for each concentration of Yoda1 and Yoda2, as shown in Figure 4C. While Yoda1 and Yoda2 both followed a similar trend, the compounds exhibited a concentration-dependent increase in slope, indicating a higher rate of hyperpolarisation at higher concentrations. Figure 4D,E display a waterfall plot of representative membrane potential traces across the concentration gradient for both activators. The traces highlight a consistent pattern of faster hyperpolarisation at higher concentrations of the reagents, as well as a longer duration of effect. Figure 5A–C aimed to determine whether biological variability was a factor to consider when analysing the data. The error bars in Figure 4B,C, therefore, reflect biological variability due to donors rather than experimental error. This was particularly evident at intermediate concentrations such as 480 nM, where individual responses to the activators diverged significantly across donors.

## 4. Discussion

At first glance, it looks easy and straightforward: an agonist or an activator of an ion channel increases its open probability in a dose-dependent manner in a particular environment/cell type at a given temperature. This means that when the whole-cell current is measured in dependence of the activator’s concentration, a so-called dose–response curve is generated for Piezo1 in RBCs upon stimulation with Yoda1 and, alternately, with Yoda2 (Figure 1). This gives characteristic EC_50_ values of 1391 nM and 305 nM for Yoda1 and Yoda2, respectively.

However, the activity of Piezo1 in RBCs is mostly associated with a Ca^2+^ influx. This is so predominant that it is sometimes overlooked that Piezo1 is a non-selective cation channel. The high Ca^2+^ gradient of 20,000 between the inside (60 nM) and the outside (1.2 mM) of the cell (under physiological conditions) and the high signalling capability of the Ca^2+^ place this ion in the centre of interest [33]. Therefore, it makes a lot of sense to consider the dose of the Yoda compounds towards the magnitude of the Ca^2+^ response in terms of fluorescence intensity of the Ca^2+^ probe Fluo-4 (Figure 2 and Figure 3). However, additional factors must now be considered:

(i) The Ca^2+^ entry, and hence the fluorescence of the dye, accumulates over time, i.e., the kinetic properties of Piezo1 or, more precisely, the kinetic modulation of Piezo1 by the different Yoda variants come into play.
(ii) There are further players inside the RBC that influence the free Ca^2+^ concentration (in a possibly Ca^2+^ concentration-dependent manner), most notably the Ca^2+^ pumps (mainly PMCA4) [34].

Figure 2C–F show that (depending on the Yoda concentrations) the RBC response increases over time, reaching a maximum (which is in addition to the strength of the response, and also temporarily different for different Yoda concentrations) and finally declines over time. Therefore, the plot of the maximal response (which is at different time points after Yoda addition; Figure 2C,D) has only limited value. Relating the patch clamp data to the Ca^2+^ measurements, it seems obvious that Yoda1 results in a prolonged or sustained opening of Piezo1 in RBCs (EC_50_ in a similar order of magnitude for patch clamp and Ca^2+^ measurements), while for the Yoda2 Ca^2+^ response, the EC_50_ is already three times the Yoda2 concentration from patch clamp recordings. Furthermore, there is a striking difference in the fraction of responding cells between patch clamp and Ca^2+^ measurements. We see two main reasons for this: Firstly, the high-throughput automated patch clamp devices are not built to measure single-channel recordings and consequently, multiple open channels are required to have a sufficient signal-to-noise ratio to classify RBCs as responding cells. In contrast, channel activity in the Ca^2+^ measurements accumulates over time, resulting in a slightly more robust read-out parameter. Secondly, in whole-cell mode in patch clamp measurements, the intracellular solution is ‘washed’ out of the cell, which may affect channel activity and is another possible explanation for the differences in response between patch clamp and Ca^2+^ measurements. However, it is not possible to estimate the specific contribution of these effects.

Going a step further, namely on the effect of increased intracellular Ca^2+^ in RBCs: A main feature in RBC volume regulation is the activation of the Gárdos channel, and this volume regulation is driven by the functional interaction between Piezo1 and the Gárdos channel. This interaction is so far seen as the major physiological function triggered by Piezo1 [7,30,35] therefore, it is worthwhile to consider the Gárdos channel activity as one of the response parameters upon Yoda dose application. A very specific read-out parameter for Gárdos channel activity in RBCs is the hyperpolarisation of the RBC membrane potential [27] and a very reliable method to measure the membrane potential in RBCs is the MBE method [29]. The result of this approach is summarised in Figure 4, and the response shows a (Yoda concentration-dependent) temporal modulation (Waterfall plots; Figure 4D,E) showing yet a different dose–response curve (Figure 4B) as the direct biophysical Yoda-dependent characterisation of Piezo1 (Figure 1E) and the Piezo1 induced Ca^2+^ entry (Figure 2B). The activation of the Gárdos channel requires Ca^2+^ concentrations that are well below the ones that can be reached by full Piezo1 activation; therefore, the EC_50_ values of the MBE measurements are below the ones for the Ca^2+^ influx. In particular, in Figure 3A, the Ca^2+^ rises quicker for Yoda1 and consequently, the threshold for the Gárdos channel activation is reached earlier compared to Yoda2. The slow onset of Ca^2+^ rise with low concentrations of the substances was obviously not long enough to reach a plateau before the Gárdos channels started to inactivate, so it might be that the slow kinetics of Yoda-induced Ca^2+^ rise is a factor that explains some of the differences seen with the other methods.

Looking for the general reasons, differences in the EC_50_ appear in the different measurement modes. We can identify several factors that can be grouped into three classes:

(i) Although all measurements relate to the activity of Piezo1, they are differently linked to the channel activity, as already discussed above. Patch clamp is the direct measurement, the Ca^2+^ increase is the response of the channel opening but modulated by other factors, and the activity of the Gárdos channel is even further downstream with the potential for further modulations.
(ii) The nature of the measurements is vastly different. While patch clamp and Ca^2+^ measurements are single-cell techniques, the MBE method is a cell population measurement presenting an average value of all cells, whereas in the patch clamp recordings, non-responding cells are not considered. In the whole-cell configuration, the intracellular compartment is connected to a reservoir containing the internal solution, resulting in a wash-out of the cytosol. In contrast, Ca^2+^ and MBE measurements are performed on intact cells, just modulated by the abundance of Fluo-4 in the cytosol or CCCP in the cell membrane, respectively.
(iii) The concrete experimental conditions can be different. This is less about different personnel or laboratory locations, but rather about the fact that different methods require different conditions. This can be the composition of the ionic solutions, e.g., patch clamp recordings require a high fluoride concentration (110 mM) for an efficient seal formation, or the nature of the MBE method requires a pH-unbuffered solution. Also, patch clamp recordings and Ca^2+^ measurements are performed at room temperature (due to historical reasons and technical limitations, respectively), whereas the MBE method needs to be performed at 37 °C. Furthermore, for all three types of measurements, the effect of the Yoda compounds is convoluted with mechanical stress on the RBCs. All three major methods applied (patch-clamp technique, flow cytometry, and MBE method) induce some but different mechanical stress to the RBCs (cell suction-induced membrane curvature change, flow in tubes and chambers, stirring of cell suspension, respectively). Although this stress was present in the control condition (without Yoda stimulation), we cannot exclude a different effect of the mechanical stress on the Yoda-induced activity of Piezo1.

All three modes contribute to the different EC_50_ values, but it would go far beyond the scope of this paper to decipher and quantify the specific contributions, still convoluted with the different kinetic actions of Yoda1 and Yoda2. Our aim is rather to raise awareness of the underlying complexity.

## 5. Conclusions

The ‘real’ EC_50_ (meaning the biophysical property) values for Piezo1 activation in RBCs are the values given in the second column of Table 1, with Yoda2 presenting a higher efficiency compared to Yoda1. However, looking for a cellular response, the picture changes and is even different in both numbers and in the relation of Yoda1 and Yoda2 to each other for the different read-outs. The reasons for these differences are multifactorial and discussed in more detail above.

As a recommendation for the choice of Yoda1 or Yoda2, it all depends on the design of the experiments: when full activation of Piezo1 is required, a condition where typically three times EC_50_ concentrations are used, 3 µM of either Yoda1 or Yoda2 should be on the safe side. However, in their activation kinetics, Yoda1 and Yoda2 show distinct differences (Figure 1, Figure 2, Figure 3 and Figure 4 and Appendix B). When Piezo1 stimulation around EC_50_ is required, the recommendation is (even when performing similar experiments as presented here) to determine the EC_50_ of the Yoda compound for the particular experimental settings and conditions (including the Yoda batch) as an experimental preparation to ensure correct concentrations because the EC_50_ values are highly sensitive on multiple parameters, as discussed above.

Along the same line of argumentation, when mutations or variants of Piezo1 are measured, i.e., when RBCs from patients with Hereditary Xerocytosis are investigated, we know that Piezo1 presents an altered activity [36,37] and hence, the EC_50_ for Yoda1 and Yoda2 will be different. Still, in an initial approach, the EC_50_ for healthy RBCs may be applied to see putative differences, but this is most likely not the EC_50_ of the Piezo1 variant. Vice versa, this makes the Yoda compounds valuable tools in investigating and characterising the RBCs of Hereditary Xerocytosis patients.

## Figures and Tables

**Table 1:** Summary of the EC_50_ data for Yoda1 and Yoda2 for all read-out methods applied.

Compound	EC_50_ (Patch Clamp) [nM]	EC_50_ (Ca^2+^ Measurements) [nM]	EC_50_ (Membrane Potential) [nM]
Yoda1	1391	1181	305
Yoda2	305	986	465

## Data Availability

The raw data supporting the conclusions of this article will be made available by the authors on request.

## Abbreviations

The following abbreviations are used in this manuscript:

a.u.	arbitrary units
DMSO	Di-Methyl-Sulf-Oxide
MBE	Macey–Bennekou–Egée
ns	not significant
RBCs	red blood cells
RCF	relative centrifugal force
